# Women on Hormone Therapy with Ischemic Stroke, Effects on Deficits and Recovery

**DOI:** 10.31531/edwiser.jnnpr.1000103

**Published:** 2019-04-01

**Authors:** Aliza Brown, Jordan Wells, Sanjeeva Onteddu, Gwendolyn Bryant-Smith, Rohan Sharma, Renee Joiner, Krishna Nalleballe, Gloria Richard-Davis, Sen Sheng, Tina Benton, William Culp, Curtis Lowery

**Affiliations:** 1 Department of Neurology, University of Arkansas for Medical Sciences, Little Rock, AR; 2 Department of Radiology, University of Arkansas for Medical Sciences, Little Rock, AR; 3 Department of Obstetrics and Gynecology, University of Arkansas for Medical Sciences, Little Rock, AR; 4 Institute for Digital Health & Innovation, University of Arkansas for Medical Sciences, Little Rock, AR

**Keywords:** Stroke, Hormone replacement therapy, Women, Telestroke

## Abstract

**Background:**

Hormone replacement therapy (HT) for post-menopausal women is associated with increased incidence of ischemic stroke risk. Effects of HT on stroke related deficits and functional outcomes in acute ischemic stroke (AIS) are uncertain. We retrospectively examined female consult data for HT use and National Institutes of Health Stroke Score (NIHSS) at baseline and recovery for 2015 and 2016 in a large stroke telemedicine program.

**Hypothesis:**

The age of women who acknowledged HT use will negatively impact stroke severity and outcomes.

**Methods:**

We analyzed consult data from two consecutive years for all women and included HT use, current age, and baseline and 24 h NIHSS’s. We included all women consults regardless of IV Alteplase treatment. 24 h NIHSS and three month modified Rankin scale (mRS) were included from women given IV Alteplase.

**Results:**

*Strokes were identified in 523 women and 244 women received Alteplase therapy. Women without HT use numbered 459 and 64 women listed HT use. Mean NIHSS scores regardless of HT use significantly improved 24 h NIHSS vs. baseline NIHSS (p<0.0001). Baseline NIHSS scores were significantly improved in women on HT vs. non-HT users (p=0.01) in women age 50 to 79 years. Although mean NIHSS scores at 24h was not different from HT to no HT use (4.9* ± *1.6 vs. 7.8* ± *0.6, p=0.08) a trend was present for lower NIHSS scores for women 50–79 years. The mRS scores at three months indicated significant improvements among HT users vs. non-HT use (1.46 ± 0.4 vs. 2.51 ± 0.2, p=0.05).*

**Conclusion:**

While cautions persist on the use, route and dosage of HT for risks of ischemic stroke, the HT moderation of AIS deficits and outcomes in women <80 years of age warrants further investigation.

## Introduction

Since Hormone therapy (HT) is still effective at relieving menopausal symptoms, there has been a large effort to determine when HT could be appropriate and understanding the balance of risk *vs.* benefit [1–3]. Understanding the balance of risk *vs.* benefit is critical since ischemic stroke is the fourth [4] leading cause of death for women.

Hormone therapy is used to encompass estrogen therapy (ET) and estrogen progesterone (EPT). Now HT is FDA approved to treat four conditions resulting from hypoestrogenia due to menopause: 1) moderate to several vasomotor symptoms 2) prevention of osteoporosis 3) vulvovaginal atrophy and 4) premature ovarian insufficiency. Historically, women were quickly directed towards HT for menopausal symptoms, such as hot flashes, as well as prevention of osteoporosis and cardiovascular disease (CVD). A study released by the landmark Women’s Health Initiative (WHI) in 2002 revealed that when HT is started late it provides no protective effects for women over 60 or over 10 years onset of menopause as well as increasing the risk of CVD and stroke [1,2].

One of the major benefits of HT is relief of menopausal symptoms in women, but another largely unknown benefit that is not as well reported, may be the neuroprotective activities of estrogen. The mechanism of action of estrogen has been elusive and appears to act in both receptor-dependent and receptor-independent mechanisms. Some of the proposed mechanisms include increased anti-apoptotic activity, increased prosurvival, as well as anti-oxidant activity in neurons [5]. These characteristics are ideal in the situation of acute ischemia. These proposed mechanisms are hypothesized to represent the positive benefits seen in recovery of acute ischemic stroke by prolonging the time neurons can survive without adequate blood flow.

With oral HT’s there has been an increased risk of ischemic stroke by about one-third which appears to be limited to ischemic stroke. Recent limited evidence suggested a lesser or ‘no risk of stroke’ with lower dose transdermal application (<50 ug/day) and a ‘reduced risk of stroke’ with vaginal HT application [1,3,6]. What remains unclear from these studies is HT use and recovery outcomes following women who experienced an ischemic stroke.

We retrospectively examined two years of consult information from the Arkansas Stroke Assistance through Virtual Emergency Support (AR SAVES) telestroke program for HT use, National Institute of Health Stroke Scale scores (NIHSS) at baseline (prior to stroke treatment) and 24 h after stroke. To determine if HT use impacted severity of stroke and recovery, the NIHSS’s at baseline and 24 h were compared NIHSS’s from the Arkansas Stroke Assistance through Virtual Emergency Support (AR SAVES) telestroke program.

## Methods

Review of retrospective de-identified data collected by AR SAVES was approved by the hub site Institutional Review Board as not human research. All data encompasses a review of January 1, 2015 to December 31, 2016. The AR SAVES program conducts monthly reviews of data for quality improvement and to support all participating spoke sites.

The AR SAVES telestroke network spans across the state using the hub and spoke model, with 54 participating community hospitals as spokes [7]. Our study included data from all female adult participants (>18 years) examined at all 54 participating community hospitals (spoke sites) in the state-based AR SAVES telestroke network. Consults positive for stroke were utilized in the data review.

Spoke sites range in bed size from 25 up to 438, with 23 sites at <50 beds. The spoke site Code Stroke practice and AR SAVES evidence-based stroke pathways is based on the time targets of the AHA’s Get-With-the-Guidelines (2014) and are provided to the rural hospitals along with on-site education. Six stroke neurologists had rotating call for consults at all 54 spoke hospitals. Spoke hospitals use mobile video carts each with a camera and computer linked to the hub center.

### Pathway and assessment:

All suspected acute stroke patients arriving in the ED were given an initial stroke assessment using the NIHSS. Following CT imagery and ED physician review, a call was made to the AR SAVES Call Center, which then contacted the attending hub neurologist on call. Following login to TMS, the stroke neurologist consultant viewed and examined patients by camera and information provided by the remote site, such as vital signs and time last seen at baseline. During the examination, the consultant would speak to the patient and/or family, perform the NIHSS (baseline), and view the computed tomographic (CT) scan that the site has previously uploaded. A decision to treat or not to treat with Alteplase was made, in concert with the on-site ED physicians, and transmitted to the spoke.

### NIHSS:

Patients were assessed for NIHSS (baseline) with scores >3 and no contraindications are considered Alteplase candidates. In addition, recommendations for additional diagnostic evaluation and therapy are provided, along with recommendations and arrangements for transfer to a higher level of care facility as appropriate. Only Alteplase therapy patients’ NIHSS’s were re-evaluated for treatment outcomes at 24 h (24 h NIHSS).

### Patient population subgroups:

Subgroups of female patient data were subdivided into decade long age groups (20s, 30s, 40s, 50s, 60s, 70s and 80s and above).

### Evaluating HT status:

Additional division of groups was achieved by HT status. The HT status was determined by the information entered by the call center as the patient was going through the consult process. Status of HT use was retrospectively determined as a simple yes or no response as recorded by the AR SAVES call center staff. A yes response was interpreted as the patient was currently on HT or was previously on HT at an earlier time point. The exact age or time of the patient on HT was not determined. Also not determined was the exact nature of the Rx for HT.

### Statistics:

The reported baseline NIHSS and 24 h NIHSS were determined for female consult patients in all age groups. Scores were compared at baseline and 24 h in no and yes HT groups using the ANOVA test. Mean scores were compared among consults among all age groups with and without HT treatment were made using Fisher’s PLSD test for post hoc analysis. Statistical significance was considered to be p<0.05.

## Results

### Female patient population:

Over the two-year period a total of 1,553 patients were seen as consults. Of these consults 842 were women and 523 were confirmed as strokes.

All data was limited to consults confirmed as positive for stroke. These included 244 consults treated with Alteplase and 279 not treated with Alteplase. Forty-seven of the non-Alteplase treated strokes had missing baseline NIHSS, leaving 232 for analysis.

All female consult data with a reported baseline NIHSS (n=476) and a 24 h NIHSS in the Alteplase treatment group (n=235) were used in this retrospective review (nine of the 244 females had missing 24 h NIHSS scores). The 235 Alteplase treated consults had significantly improved 24 h NIHSS *vs.* baseline NIHSS in a paired t-test (7.5 +/− 0.5 vs. 11.2 +/− 0.5, p<0.0001).

### Age:

An age range of each decade in 20’s, 30’s, 40’s, 50’s, 60’s, 70’s and 80’s and above was used to further categorize the female consults. Women with a reported baseline NIHSS (n=476) numbered 4, 18, 49, 84, 104, 99 and 118 in the seven age groups. Neurological deficits (NIHSS) at baseline was evaluated for all women consults by age range regardless of HT use. Age was significantly related to higher baseline and 24 h NIHSS’s at p<0.0001 and p=0.0023, respectively.

### Age, Baseline NIHSS:

**Figure 1:**
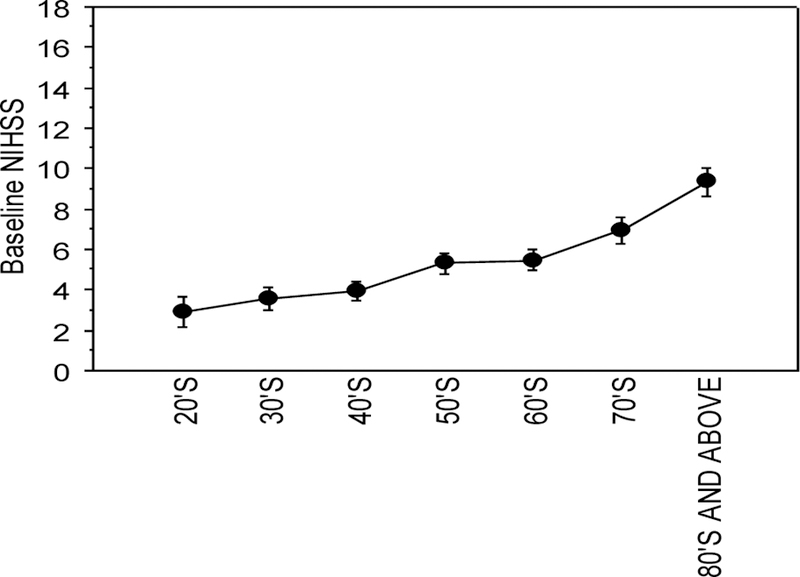
Baseline NIHSS for all women consults by age range. All women consult regardless of stroke treatment. Significance of lowered NIHSS were observed for women in the 20’s to 70’s *vs.* 80’s and above, p ≤ 0.0068. Mean NIHSS ± sem are reported.

All women consult in their 80’s and above age ranges had significantly higher baseline NIHSS *vs.* women in their 20’s, 30’s, 40’s, 50’s and 70’s, at p<0.0068, respectively (Figure 1).

### HT use:

#### HT Use x Age x Baseline NIHSS.

**Figure 2:**
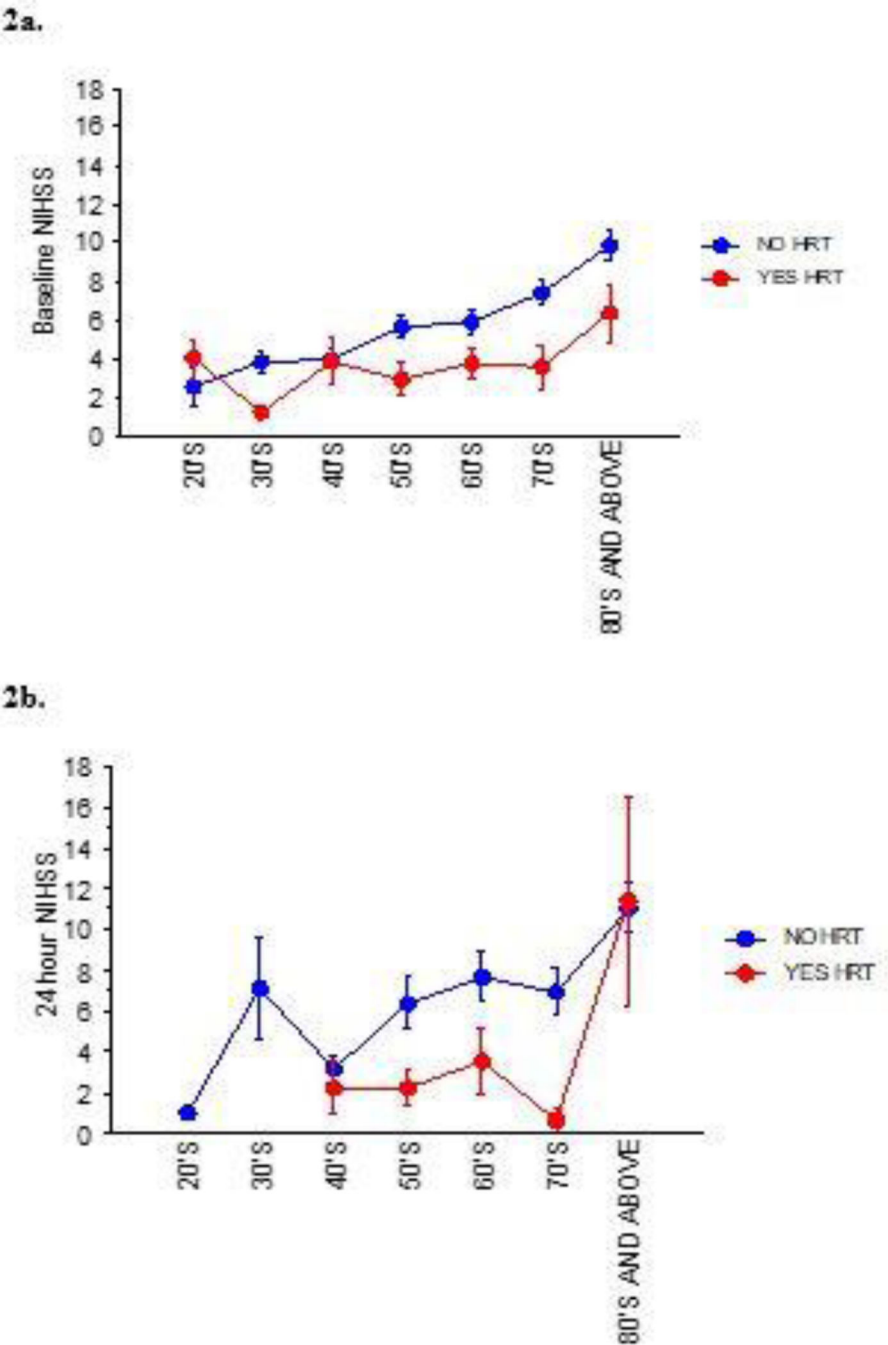
Baseline and 24 h NIHSS for Alteplasetreated women consults with and without HT use by age range. Panel 2a-All women consults treated with Alteplase are included in this analysis. Women who reported HT use indicated lower baseline NIHSS’s in the 50’s, 60’s and 70’s vs. non-HT users at p<0.01 Panel 2b-Women on HT were not different from non-HT users at p=0.08. Mean NIHSS ± sem are reported.

A total of 64 women had affirmative entries for HT use and 459 under no HT use (n=523). There were greater numbers of women in the 40’s and above age groups for HT use in the respective age range at 3, 0, 7, 8, 18, 13 and 15, respectively (Figure 2a). The baseline NIHSS’s when divided into specific age ranges showed decreased scores when on HT *vs.* no HT use from 50s through 70s, p<0.01.

### HT use:

#### *HT Use x Age x* 24 h *NIHSS x Alteplase therapy*.

All 235 Alteplase-treated female consults’ 24 h NIHSS’s shown in Figure 2b, numbered 1, 7, 19, 42, 56, 47 and 63 in the seven age groups. A higher NIHSS was observed in the 80’s and above age group *vs.* 40’s, 50’s, 60’s and 70’s, although there was no significance at p<0.08.

### NIHSS’s:

#### HT Use x Baseline NIHSS.

**Figure 3:**
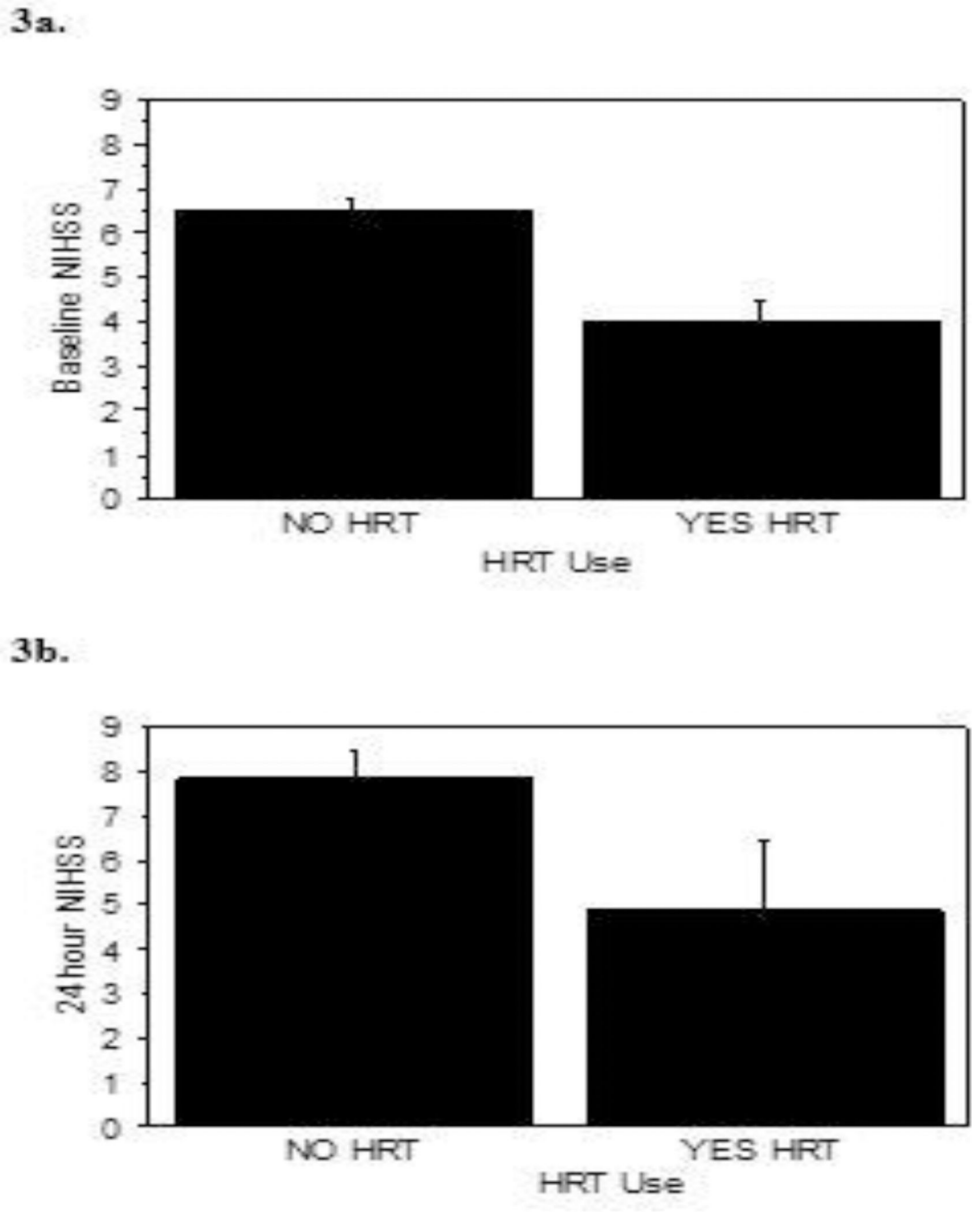
Overall baseline and 24 h NIHSS for all women consults. Panel 3a-All women consults with HT use reported indicated a lower baseline NIHSS, p=0.01. Panel 3b-Consults treated with Alteplase with and without HT use were compared at 24 h, p=0.08. Mean NIHSS ± sem are reported.

Of the 124 HT users, there were 111 with reported baseline NIHSS and 627 by non-HT users. Overall age groups, the HT users had lower NIHSS’s at baseline *vs.* non-HT users 5.5 ± 0.7 *vs.* 8.4 ± 0.4 (p=0.01), respectively (Figure 3a).

### NIHSS’s:

#### HT Use x 24 h NIHSS.

The 24 h NIHSS estimated for the 244 Alteplase treated women, there were 27 HT users and 208 non-HT users. There were no differences between this HT use and non-use groups at 4.9 *vs.* 7.8, respectively at p=0.08 (Figure 3b).

### Baseline *vs*. 24 hours:

**Figure 4:**
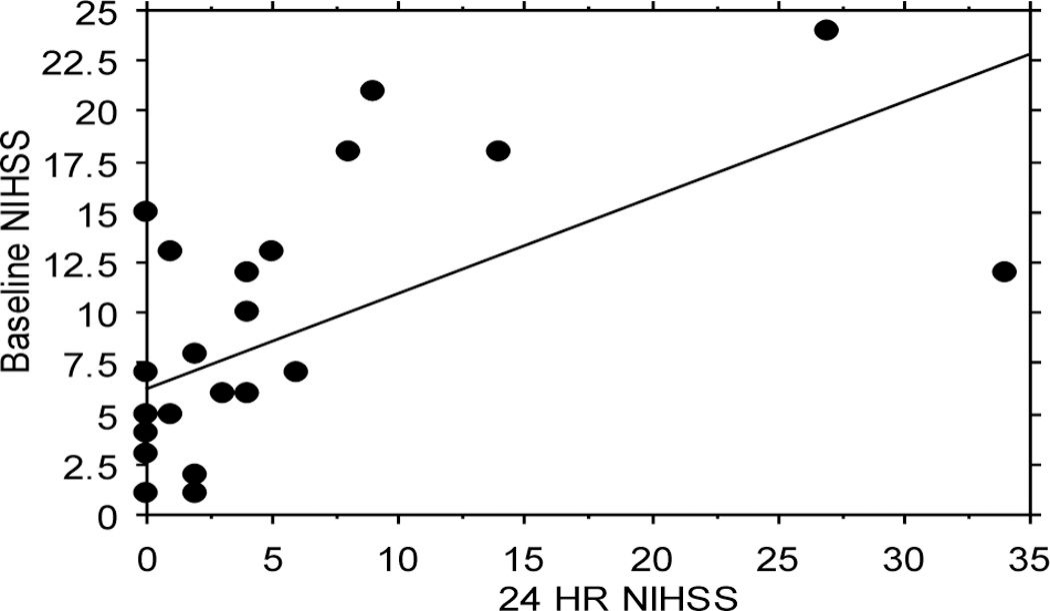
Regression analysis of baseline NIHSS *vs*. 24 h NIHSS. All 27 women consults who received Alteplase and reported HT use had baseline *vs.* 24 h NIHSS positively correlated, R=0.602, p=0.0009.

In a pairwise t-test comparison of the 27 HT user consult baseline NIHSS’s *vs.* their 24 h NIHSS, the critical difference between means was 3.7 with a p<0.0001. The 27 HT users had a 93% improvement in 24 h NIHSS scores (^Max^ Baseline NIHSS score vs. ^Max^ 24 h NIHSS score, 22 vs. 15, respectively) (Fig. 4). Additionally, 22 HT users (81%) reported a lower range of ^Max^ Baseline NIHSS scores *vs.* ^Max^ 24 h NIHSS scores (15 *vs.* 6, respectively) (Figure 4). Women on HT had significantly improved 24 h NIHSS vs. baseline, (4.9 ± 1.6 vs. 8.4 ± 1.2, p=0.0009).

### 3-month mRS.

#### 3-month outcomes x HT:

In a comparison of HT *vs.* non-HT use in a 3-month mRS, the HT users reported better outcomes *vs*. non-HT (n=13 and =120, respectively, 1.46 ± 0.4 vs. 2.51 ± 0.2, p=0.05).

## Discussion

The age at which the timing of HT administration began in the women treated for stroke while unknown is an important consideration of HT’s risks and benefits. Systemic hormone therapy is usually initiated in symptomatic women within 10 years of menopause. Women may experience menopausal symptoms 5–10 years before the final menstrual period and up to 7 years’ average post menopause [8]. Continuation of HT beyond 5 years requires reassessment and individualization of treatment. For women experiencing premature menopause or premature ovarian insufficiency (POI), natural or surgical, therapy should be started at age of onset and managed accordingly after the average age of menopause. For women over age 60 or >10 years post menopause, it is ill advised to start therapy. However, therapy may be continued after assessment, if benefits out-weigh risks. In our study, the distribution of women by decade using hormone therapy is consistent with these recommendations. Most women on HT were >40 years old. Those less than 40 on therapy were likely to have experienced premature menopause.

Late initiation of HT increases the risk of CVD and stroke. Additionally, many observational and randomized controlled trials have also shown an increased incidence of invasive breast cancer (HR=1.28) when combined estrogen and progestin HT are used in postmenopausal women [1,9]. However, in the estrogen only arm breast cancer risks were reduced. (HR=0.7) [9]. The WHI randomized controlled trial also reported the cancer association in 2002 [1]. A variety of hormone regimens are now available for eradication of menopausal symptoms. However, additional studies now suggest, that not all HT have the same level of breast cancer risk [10]. Fournier et al. [10], in a study in 2008, showed that the choice of progestin used in combined HT played a significant role in the overall increase in breast cancer risk. The length of time spent on HT also appears to effect overall breast cancer risk. The Collaborative Group on Hormonal Factors in Breast Cancer [11] reanalyzed data and reported an increased breast cancer risk in patients on combined therapy of more than 5 years. A study by Chen et al. [12] estimated that 349 women per 100,000 ages 60–69 would develop breast cancer after 5 years of recent HT use compared to 230 per 100,000 in non-users. The benefits versus risks should be evaluated for each patient considering HT [13,14].

Elevated estrogen levels increase coagulation factors, which may precipitate acute myocardial infarctions or ischemic strokes. Current HT guidelines recommend using the lowest effective dose to control symptoms and minimize adverse effects. At lower doses of estrogen, serum levels of estrogen are usually in physiologic range. Transdermal or other non-oral route estrogens are theoretically safer, as they avoid the first pass hepatic effect responsible for elevating coagulation factors [15]. Elevated estrogen levels increase coagulation factors, which may precipitate acute myocardial infarctions or ischemic strokes. However, there are no head to head comparison studies [2]. Oral preparations are the most commonly prescribed hormone therapy due to reduced cost. It would, therefore, be important to know the initiation time of HT use and preparation type used in our study population.

Post-stroke 24 h NIHSS comparisons among all women, were not different in HT users *vs.* non-users (p=0.084). Trend differences in NIHSS’s were not evident until age groupings were identified. Similarly, the Women’s Estrogen for Stroke Trial (WEST) completed in 2006, also found that HT users *vs.* non-users were not different [16]. In addition, the HT users had slightly worse NIHSS scores but did not analyze the results by age groupings. In this study we observed a greater number of women stroke patients in the 60’s, 70’s and 80’s and above age categories. In addition to the higher incidence of stroke in older women the strokes were associated with a higher neurological deficit in 70’s and 80’s and above *vs.* women in their 20’s to 50’s at baseline (p<0.0068) (Figure 1). At 24 h following stroke treatment, NIHSS’s were significantly greater in the >80’s and above age group *vs.* women in their 40’s to 70’s, p<0.0068 (Figure 2).

Consistent evidence from clinical trials and observational research [17,18] indicates that standard-dose hormone therapy increases stroke risk for postmenopausal women by about one-third; increased risk may be limited to ischemic stroke. These studies report that risk is not modified by age of hormone initiation or use, or by temporal proximity to menopause, and risk is similar for estrogen plus progestogen and for unopposed estrogen. Limited evidence implies that lower doses of transdermal estradiol (≤ 50 µg/day) may not alter stroke risk. However, for women less than 60 years of age, the absolute risk of stroke from standard-dose hormone therapy is rare, about two additional strokes per 10 000 person-years of use. Consistent with this study the reported absolute risk is considerably greater for older women.

Consistent evidence from clinical trials and observational research [17,18] indicates that standard-dose hormone therapy increases stroke risk for postmenopausal women by about one-third; increased risk may be limited to ischemic stroke. These studies report that risk is not modified by age of hormone initiation or use, or by temporal proximity to menopause, and risk is similar for estrogen plus progestogen and for unopposed estrogen. Limited evidence implies that lower doses of transdermal estradiol (≤ 50 µg/day) may not alter stroke risk. However, for women less than 60 years of age, the absolute risk of stroke from standard-dose hormone therapy is rare, about two additional strokes per 10 000 person-years of use. Consistent with this study the reported absolute risk is considerably greater for older women.

## Limitations:

Limitations of our study includes the small sample size and unavailability of non-Alteplase consults in the telemedicine program. Similarly, data was not available regarding age beginning HT and length of time on therapy. Also, not available was the age of menopause and if the women were taking only estrogen or combined estrogen and progesterone pills.

## Conclusion

In conclusion, in this retrospective study though all women improved following thrombolysis with Alteplase, an age specific effect was noted on severity of stroke as indicated by baseline NIHSS and recovery as indicated by 24 h NIHSS and mRS.
